# Nonrestorative sleep is a risk factor for metabolic syndrome in the general Japanese population

**DOI:** 10.1186/s13098-023-00999-x

**Published:** 2023-02-21

**Authors:** Yuichiro Otsuka, Yoshitaka Kaneita, Katsutoshi Tanaka, Osamu Itani, Yoshiyuki Kaneko, Masahiro Suzuki, Yuuki Matsumoto, Kenichi Kuriyama

**Affiliations:** 1 Division of Public Health, Department of Social Medicine, Nihon University School of Medicine, 30-1 Oyaguchi-Kamimachi, Itabashi-Ku, Tokyo, 173-8610 Japan; 2 Department of Occupational Mental Health, Graduate School of Medical Sciences, Kitasato University, 1-15-1 Kitasato, Minami, Sagamihara 252-0374 Japan; 3 Department of Psychiatry, Nihon University School of Medicine, 30-1 Oyaguchi-Kamimachi, Itabashi-Ku, Tokyo, 173-8610 Japan; 4 Department of Sleep-Wake Disorders, National Center of Neurology and Psychiatry, National Institute of Mental Health, 4-1-1 Ogawa-Higashi, Kodaira, Tokyo 187-8553 Japan

**Keywords:** Cardiovascular risk, Diabetes, Hypertension, Metabolic syndrome, Sleep

## Abstract

**Background:**

This longitudinal study aimed to investigate the effects of nonrestorative sleep on developing metabolic syndrome (MetS) and related diseases in a general Japanese middle-aged population.

**Methods:**

Overall, 83,224 adults without MetS (mean age: 51.5 ± 3.5 years) from the Health Insurance Association in Japan were followed up for a maximum of 8 years between 2011 and 2019. The Cox proportional hazard method was used to determine whether nonrestorative sleep, assessed using a single-item question, was significantly associated with the respective development of MetS, obesity, hypertension, diabetes, and dyslipidemia. The MetS criteria were adopted by the Examination Committee for Criteria of Metabolic Syndrome in Japan*.*

**Results:**

The mean follow-up duration was 6.0 years. The incidence rate of MetS was 50.1 person-years/1,000 during the study period. Data suggested that nonrestorative sleep was associated with MetS (hazard ratio [HR]: 1.12, 95% confidence interval [CI]: 1.08–1.16) and other disorders, such as obesity (HR: 1.07, 95% CI: 1.02–1.12), hypertension (HR: 1.07, 95% CI: 1.04–1.11), and diabetes (HR: 1.07, 95% CI: 1.01–1.12) but not with dyslipidemia (HR: 1.00, 95% CI: 0.97–1.03).

**Conclusions:**

Nonrestorative sleep is associated with the development of MetS and many of its core components in the middle-aged Japanese population. Therefore, assessing nonrestorative sleep may help identify individuals at a risk of MetS development.

**Supplementary Information:**

The online version contains supplementary material available at 10.1186/s13098-023-00999-x.

## Background

Metabolic syndrome (MetS) represents a multicomponent risk factor for cardiovascular disease (CVD) and type 2 diabetes, including abdominal (visceral) obesity, high blood pressure, high fasting plasma glucose levels, elevated triglyceride (TG) levels, and low high-density lipoprotein cholesterol (HDL-C) levels [1]. However, several studies have shown that MetS is associated with the risk of cancer, depression, and all-cause mortality, in addition to CVD [2–4]. Therefore, effective public health strategies to control and treat MetS are necessary.

Several lifestyle factors, including unhealthy dietary habits, low physical activity, and smoking, have been associated with the development of MetS [1]. Additionally, sleep complications are positively associated with the development of MetS. For example, a recent meta-analysis reported that short sleep duration was significantly associated with the risk of developing MetS [5]. Other meta-analyses have shown that insomnia is positively associated with the development of MetS-related symptoms, including hypertension, hyperglycemia, and obesity [6]. Furthermore, poor sleep quality is significantly associated with the development of MetS [7].

Recently, nonrestorative sleep (NRS) has been recognized as a major sleep problem, in addition to insomnia, sleep-disordered breathing (SDB), and short sleep duration [8]. NRS is a subjective experience of unrefreshing sleep [9], and its prevalence ranges from 2.4 to 42% worldwide [8]. In addition, NRS is associated with mental health problems, including depression [10]. In the general population, increasing evidence indicates an association between NRS and developing metabolic-related diseases [11–15]. Previous studies have shown that NRS is associated with obesity, diabetes mellitus, and coronary disease [11–15]. However, most of these studies had cross-sectional designs, which prevented the determination of causal associations. A longitudinal study comprising 2291 middle-aged participants in Hong Kong found that baseline NRS was significantly associated with the development of diabetes mellitus, rather than hypertension, at follow-up [13]. However, to our knowledge, only a few longitudinal studies have evaluated the effects of NRS on incident MetS. In addition, NRS has been assessed as one symptom under the umbrella category of insomnia. Consequently, the effects of NRS alone remain largely unclear.

Therefore, we conducted a retrospective cohort study to assess the effect of NRS on incident MetS in the general adult population of Japan.

## Methods

### Ethics approval

The Ethics Committee of the Nihon University School of Medicine approved the protocol for this study (Approval No. 2021-07), which was conducted in accordance with the Declaration of Helsinki. The need for patient consent was waived because of the use of unidentifiable individual data.

### Participants

This study used data from a retrospective cohort study that included participants who underwent an annual health checkup facilitated by the Health Insurance Association for Architecture and Civil Engineering Companies in Japan. In Japan, by law, employees should receive health checkups at least once a year. The participants were employees working for architecture and civil engineering companies in Japan and their family members. A baseline survey was conducted between April 2011 and March 2012 (N = 135,609). The survey was completed in March 2019.

### Inclusion and exclusion criteria

We included participants aged between 39 and 75 years at the beginning of the study (the baseline), who were asked about NRS and lifestyle. In contrast, we excluded participants who did not answer the NRS questions since it was not mandatory to do so during health checkups. Subsequently, we independently excluded participants with baseline MetS, obesity, hypertension, diabetes mellitus, and dyslipidemia to create corresponding cohorts.

### Definitions of various metabolic diseases

#### MetS

MetS was defined based on the diagnostic criteria of the Japanese Committee to Evaluate Diagnostic Standards for Metabolic Syndrome [16]. In this study, the following were used as diagnostic criteria for MetS: abdominal obesity, defined as a waist circumference of ≥ 85 cm and ≥ 90 cm in men and women, respectively, and with two or more of the following conditions being met: (a) systolic and diastolic blood pressure measurements of ≥ 130 mmHg and 85 mmHg, respectively, or use of antihypertensive medication; (b) plasma triglyceride (TG) of ≥ 150 mg/dL, HDL cholesterol of < 40 mg/dL, or use of antilipidemic medication; and (c) plasma glucose of ≥ 110 mg/dL or use of hypoglycemic medication.

#### Obesity

Obesity was defined as a body mass index (BMI) of ≥ 25 kg/m^2^ based on the standards of the Japan Society for the Study of Obesity [17].

#### Hypertension

According to the criteria of the Japanese Society of Hypertension, participants were identified as having hypertension when their systolic blood pressure was ≥ 140 mmHg or their diastolic blood pressure was ≥ 90 mmHg [18]. Participants who self-reported using antihypertensive drugs were classified as having hypertension.

#### Diabetes mellitus

Participants were identified as having diabetes when their fasting plasma glucose level was ≥ 126 mg/dL (≥ 7.0 mmol/L), or their glycated hemoglobin (HbA1c) level was ≥ 6.5% (≥ 48 mmol/mol), according to the criteria of the Japan Diabetes Society [19]. Participants who self-reported using antihyperglycemic drugs were also defined as having diabetes mellitus.

#### Dyslipidemia

Based on the criteria of the Japan Atherosclerosis Society, participants were considered to have dyslipidemia if plasma HDL-C, or TG levels were < 40 mg/dL, or ≥ 150 mg/dL, respectively [20]. Participants who self-reported using lipid-lowering drugs were also defined as having dyslipidemia.

#### NRS

NRS was used as the exposure variable. Information on NRS was obtained by asking participants: “Do you feel refreshed after a typical night’s sleep?” The participants could only answer “yes” or “no.” NRS was defined when the patient answered “no [12].”

### Covariates

At each survey point, basic characteristics (age and sex) and lifestyle factors were measured. Lifestyle factors included the following: non-regular exercise (“I exercised twice or more per week for ≥ 30 min over the past 1 year or more”: no/yes), skipping breakfast (I skip breakfast at least three times a week: yes/no), habitual smoking (“I smoked ≥ 100 cigarettes over 6 months and smoked in the previous month”: yes/no), and heavy consumption of alcohol (“every day and ≥ 40 g per day” and “occasionally and ≥ 60 g per day”). In addition, alcohol intake was assessed using the following two questions: “How often do you drink alcoholic beverages? (every day, occasionally, or rarely/never) and “How many alcoholic beverages do you drink on the days you drink? (< 1 drink per day, 1–2 drinks per day, 2–3 drinks per day, ≥ 3 drinks per day). Approximately 500 mL beer, 80 mL “shochu” [a Japanese liquor similar to vodka], 60 mL whiskey, or 240 mL wine was assumed to be one standard drink, with each drink containing 20 g of ethanol.

### Missing data

Missing values were imputed by multiple imputations using the chain equation method during the follow-up period [21], and 10 sets of imputed data were generated.

### Statistical analyses

First, baseline characteristics are described using means (% and 95% confidence interval [CI]) and counts (n) for continuous and categorical variables, respectively. Second, the incidence rates of MetS and related diseases were calculated. Third, the effects of incident MetS on NRS were explored using Cox proportional hazards regression to estimate the hazard ratios (HR) and their 95% CI. The covariates were age, sex, BMI, and lifestyle factors, including non-regular exercise, skipping breakfast, habitual smoking, and heavy alcohol consumption. We used factors associated with MetS in a previous study to determine these variables [22]. However, these variables were considered time-dependent since each individual’s NRS and lifestyle factors may have changed throughout the study period. Fourth, we used Cox proportional hazards regression to investigate the effect of incident MetS-related diseases (obesity, hypertension, diabetes, and dyslipidemia). Finally, we evaluated the differences between sexes using the same Cox regression for the sensitivity analysis. All analyses were performed using Stata version 17.0 (StataCorp, College Station, TX, USA). All tests were two-tailed, with statistical significance set at a P-value of < 0.05.

## Results

### Participant characteristics

**Fig. 1:**
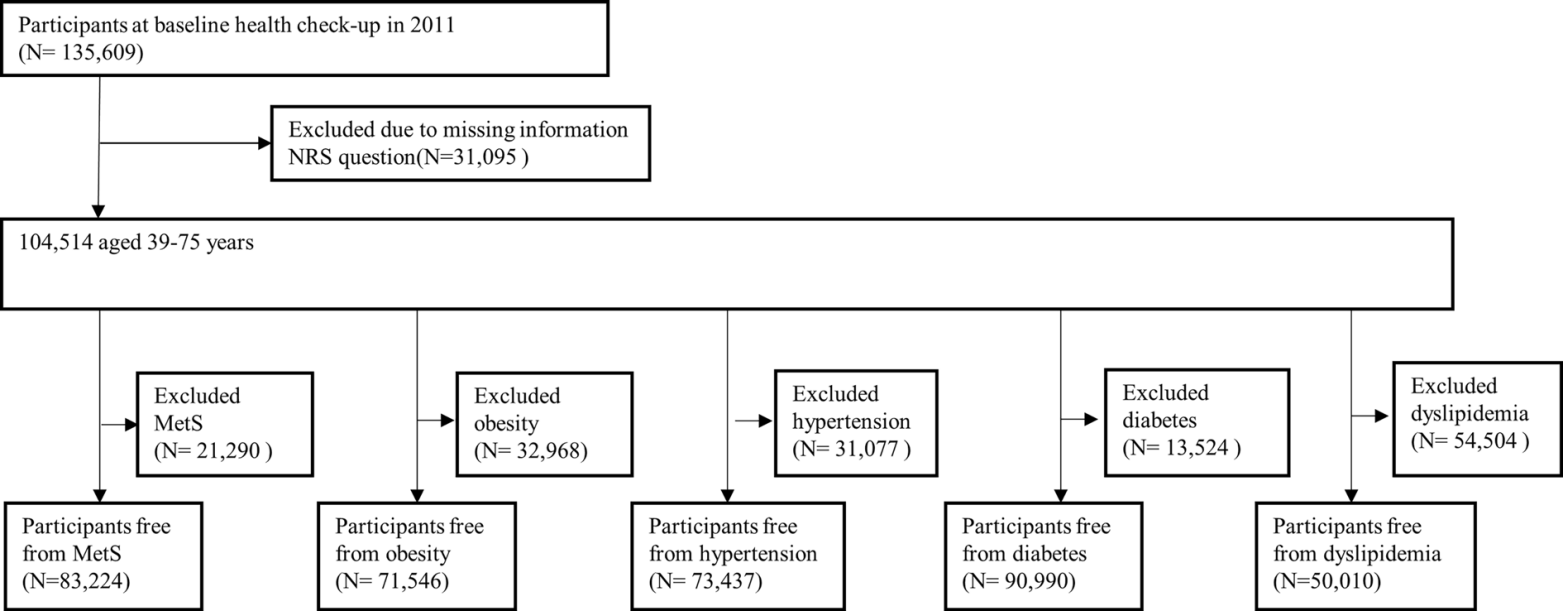
Flowchart of participant selection. MetS, metabolic syndrome

Figure 1 shows the flowchart for participant selection. In the baseline survey, 135,609 participants were screened. Participants who did not answer the NRS question were excluded because it was not mandatory to do so during health checkups. The 104,514 remaining individuals were aged 39–75 years. Furthermore, we selected participants without MetS at baseline (N = 83,224) and those without each metabolic-related disease as subsamples at baseline, including obesity (N = 71,546), hypertension (N = 73,437), diabetes (N = 90,990), and dyslipidemia (N = 50,010).

Table 1 shows the baseline characteristics of the participants for each metabolic state. In particular, similar distributions were observed in each cohort. The median age was approximately 50 years, and approximately two-thirds of the patients were male, with a median BMI of 22–23 kg/m^2^. Furthermore, 15.2% of the participants skipped breakfast, 75.3% engaged in less physical activity, 25.7% were current smokers, and 12.4% were heavy drinkers.

**Table 1:** Baseline characteristics

	Participants free from MetS (N = 83,224)		Participants free from obesity (N = 71,546)		Participants free from hypertension (N = 73,437)		Participants free from diabetes (N = 90,990)		Participants free from dyslipidemia (N = 50,010)	
NRS	34,483	41.4	29,603	41.4	32,250	43.9	39,277	43.2	21,224	42.4
Age (years)	51.5	44.5–58.5	52.0	44.7–58.9	49.7	43.8––57.0	51.3	44.4–58.2	50.2	43.9–57.8
Male sex	54,962	66.0	46,321	64.7	49,284	67.1	64,309	70.7	32,721	65.4
BMI	22.7	20.8–24.6	22.1	20.5–23.5	22.8	20.8–25.0	23.3	21.2–25.4	22.4	20.4–24.5
Hypertension	18,418	22.1	16,103	22.5	–	–	24,644	27.1	11,511	23.0
Diabetes	4117	4.9	3915	5.5	3944	5.4	–	–	2591	5.2
Dyslipidemia	37,198	44.7	31,760	44.4	34,933	47.6	45,879	50.4	–	–
Skipping breakfast	12,654	15.2	10,800	15.1	12,222	16.6	14,709	16.2	7296	14.6
Low physical activity	62,688	75.3	53,868	75.3	56,242	76.6	69,619	76.5	37,683	75.4
Current smoking	21,422	25.7	18,771	26.2	20,517	27.9	24,954	27.4	12,445	24.9
Heavy alcohol drinking	10,308	12.4	8,997	12.6	8,617	11.7	12,645	13.9	6,657	13.3

Data are expressed as n (%) or median (upper and lower quartiles)

MetS, metabolic syndrome; BMI, body mass index

### Association between NRS and MetS

Among the 83,224 participants without MetS at baseline, 17,480 developed MetS, with a mean follow-up period of 6.0 years (incidence rate, 50.1 [95% CI: 49.3–50.8] per 1,000 person-years) (Table 2). Table 3 shows the results of the Cox proportional hazard models for incident MetS. NRS was significantly associated with MetS risk in both models (Model A, HR: 1.14, 95% CI: 1.10–1.18; Model B, HR: 1.12, 95% CI: 1.08–1.16). In the sensitivity analysis, MetS HR for both sexes indicated similar trends to the original results, and there were no significant sex differences in the association between the NRS score and MetS (Additional file 1: Table S1).

**Table 2:** Incidence of MetS and related diseases

Incidence	N	Mean follow-up	Person-years/1000	95% CI
MetS	83,224	6.0	50.1	49.3–50.8
Obesity	71,546	6.0	29.1	28.5–29.7
Hypertension	73,437	6.1	63.4	62.5–64.3
Diabetes	90,990	6.1	13.7	13.4–14.1
Dyslipidemia	50,010	6.1	124.8	123.2–126.5

MetS: waist circumference of ≥ 85 cm in men and ≥ 90 cm in women and two or more of the following: (1) blood pressure of ≥ 130/85 mmHg or use of antihypertensive medication; (2) TG level of ≥ 150 mg/dL, HDL-C level of < 40 mg/dL, or use of antilipidemic medication; and (3) glucose level of ≥ 110 mg/dL or use of hypoglycemic medication

Obesity: BMI of ≥ 25 kg/m^2^

Hypertension: ≥ 140/90 mmHg or use of antihypertensive medication

Diabetes: ≥ 126 mg/dL, HbA1c level of 6.5%, or use of hypoglycemic medication

Dyslipidemia: TG level of ≥ 150 mg/dL, low HDL-C level of < 40 mg/dL, or use of antilipidemic medication

BMI, body mass index; CI, confidence interval; HDL-C, high-density lipoprotein cholesterol; MetS, metabolic syndrome; TG, triglyceride

**Table 3:** Associations between NRS and developing MetS

	Model A				Model B			
	**HR**	95% CI	*P*-value	*z*	HR	95% CI	*P*-value	*z*
NRS	1.14	1.10–1.18	< 0.001	7.2	1.12	1.08–1.16	< 0.001	6.5

Model A: Adjusted for age and sex

Model B: Adjusted for age, sex, smoking, heavy alcohol consumption, skipping breakfast, and non-regular exercise

HRs and P-values were calculated using the Cox proportional hazards model

MetS: waist circumference of ≥ 85 cm in men and ≥ 90 cm in women, and two or more of the following: (1) blood pressure of ≥ 130/85 mmHg or use of antihypertensive medication; (2) TG level of ≥ 150 mg/dL, HDL-C level of < 40 mg/dL, or use of antilipidemic medication; and (3) glucose level of ≥ 110 mg/dL or use of hypoglycemic medication

CI, confidence interval; HDL, high-density lipoprotein; HR, hazard ratio; MetS, metabolic syndrome; NRS, nonrestorative sleep; TG, triglyceride

### Association between NRS and each metabolic disease

The incidence rates of MetS-related diseases per 1000 individuals were 29.1 (95% CI: 28.5–29.7), 63.4 (95% CI: 62.5–64.3), 13.7 (95% CI: 13.4–14.1), and 124.8 (95% CI: 123.2–126.5) for obesity, hypertension, diabetes, and dyslipidemia, respectively (Table 2).

Table 4 shows the results of the Cox proportional hazard model for each metabolic-related disease. NRS was significantly associated with the risks of obesity (HR: 1.07, 95% CI: 1.02–1.12), hypertension (HR: 1.07, 95% CI: 1.04–1.11), and diabetes (HR: 1.06, 95% CI: 1.00–1.12).

**Table 4:** Associations between NRS and developing obesity, hypertension, diabetes, and dyslipidemia

	Obesity				Hypertension			
	HR *^1^	95% CI	*P-*value	*z*	HR *^2^	95% CI	*P*-value	*z*
NRS	1.07	1.02–1.12	0.003	3.0	1.07	1.04–1.11	< 0.001	4.5

	Diabetes				Dyslipidemia			
	HR *^3^	95% CI	*P-*value	z	HR *^4^	95% CI	*P-*value	z
NRS	1.07	1.01–1.12	0.017	2.4	1.00	0.97–1.03	0.910	0.1

*1 Adjusted for age, sex, smoking, heavy alcohol consumption, skipping breakfast, non-regular exercise, diabetes, hypertension, and dyslipidemia

*2 Adjusted for age, sex, BMI, smoking, heavy alcohol consumption, skipping breakfast, non-regular exercise, diabetes, and dyslipidemia

*3 Adjusted for age, sex, BMI, smoking, heavy alcohol consumption, skipping breakfast, non-regular exercise, hypertension, and dyslipidemia

*4 Adjusted for age, sex, BMI, smoking, heavy alcohol consumption, skipping breakfast, non-regular exercise, diabetes, and hypertension

HRs and P-values were calculated using the Cox proportional hazards model

Obesity: BMI of ≥ 25 kg/m^2^

Hypertension: ≥ 140/90 mmHg or use of antihypertensive medication

Diabetes: ≥ 126 mg/dL, HbA1c level of 6.5%, or use of hypoglycemic medication

Dyslipidemia: TG level of ≥ 150 mg/dL, low HDL-C level of < 40 mg/dL, or use of antilipidemic medication

BMI, body mass index; CI, confidence Interval; HDL-C, high-density lipoprotein cholesterol; HR, hazard ratio; NRS, nonrestorative sleep; TG, triglyceride

Generally, these results were consistent with the incidence rates of obesity and dyslipidemia in the subgroups analyzed according to sex, whereby NRS was associated with the risk of obesity among both men and women. However, NRS was not significantly associated with hypertension in women or diabetes in either sex (Additional file 1: Table S2).

## Discussion

To our knowledge, this is the first longitudinal study to evaluate the association between NRS and incident MetS in the general Japanese population. This study included many participants who were followed up for a maximum of 7 years. The main findings were as follows: (1) NRS was a risk factor for developing MetS; (2) NRS was also a risk factor for developing MetS-related diseases, including obesity, hypertension, and diabetes; and (3) NRS was not a risk factor for developing dyslipidemia. These findings may help develop more effective prevention strategies for MetS and its related diseases.

### NRS assessment

Several assessment methods for NRS are available [8]. Because NRS was defined as a symptom of insomnia but was excluded from the recent definition of insomnia, it was considered an independent symptom in research and was therefore included inconsistently across different studies on this topic. Therefore, it is difficult to compare the current studies because NRS measurement is not yet standardized.

### NRS and MetS

Our data revealed the association of NRS with obesity, elevated blood pressure, and worsening glucose metabolism, all of which are components of MetS. Therefore, combining these metabolic states suggests a strong association between the NRS score and MetS. Interestingly, several cross-sectional studies and a longitudinal study supported our results. For example, a single 3-year cohort study of 812 middle-aged individuals only found NRS, difficulties initiating sleep, and loud snoring to be associated with an increased risk of incident MetS [22]. Regarding cross-sectional studies, a United States cross-sectional study of 210 middle-aged individuals demonstrated that poorer sleep quality, evaluated using the Pittsburgh Sleep Quality Index, was associated with higher odds of having MetS [23]. A Chinese cross-sectional study of 1252 individuals showed that insomnia symptoms, including NRS, were associated with MetS-related conditions, such as high blood pressure, high TG level, and low HDL-C level [24]. Although previous studies have used NRS as a sleep problem for sub-analysis, this study treated NRS as the main explanatory variable. Moreover, our study involved an 8-year follow-up period of a large cohort, which strengthened the statistical validity of our data and allowed us to better explore the causal relationship between NRS status and MetS incidence in a middle-aged population.

### NRS and obesity

This study indicates that NRS significantly promoted the onset of obesity. A 7-year cohort study of 815 adults without obesity revealed that poor sleep, including NRS, predicted incident obesity after adjusting for confounders [25]. Furthermore, a cross-sectional study of 118 individuals without SDB showed that NRS was significantly more frequent in patients with obesity than in those with normal weight [11]. Therefore, NRS is likely a risk factor for obesity, as well as short sleep duration and poor sleep quality [26].

### NRS and hypertension

Consistent with our findings, two previous studies have shown that NRS is associated with the development of hypertension [27, 28]. The former used a dichotomous questionnaire to ask about the presence or absence of NRS symptoms. The latter defined NRS as experiencing mild to severe related symptoms within the past 1 year or less. However, a 6-year cohort study involving 8,757 participants demonstrated that the combination of difficulty falling asleep, staying asleep, and NRS was not associated with an increased risk of hypertension [29]. In this study, NRS was identified as a symptom of insomnia; however, its frequency was not assessed. The discrepancies in these results may be explained by methodological differences, including NRS assessments and confounding factors between studies.

### NRS and diabetes

Consistent with our cohort data, NRS was associated with diabetes in a cross-sectional study of 14,476 general Japanese [12] and in cohort studies of the Chinese population [13] and French employees [27]. However, a meta-analysis revealed that poor sleep quality and sleep duration were associated with worsening glycemic control [30]. These findings indicate that NRS is a risk factor for diabetes as well as sleep duration and poor sleep quality. 

### NRS and dyslipidemia

Our data suggest that NRS is not a risk factor for dyslipidemia. Previous studies have reported associations between sleep problems and lipid profile; however, their results were inconsistent [31, 32]. Consistent with our results, a cross-sectional study of the general Chinese population showed that insomnia symptoms accompanied by NRS were not associated with the development of dyslipidemia [31]. However, a longitudinal cohort study in Finland found that disturbed sleep, including NRS, was associated with dyslipidemia rather than short and long sleep duration [32]. The differences may be due to the study design, definitions of sleep problems and/or dyslipidemia, and adjustment for confounding factors, including sleep duration and SDB.

### Mechanism linking NRS and MetS

Multiple pathways are suggested to mediate the relationships between sleep disturbance, MetS, and MetS components [33]. First, insufficient sleep is assumed to affect energy balance through increased regulation of appetite and frequency of eating and reduced energy expenditure [30]. Sleep disruption has been associated with altered leptin levels and resistance, leading to the dysregulation of the hypothalamic–pituitary–adrenal axis system, thereby leading to elevated glucose levels and weight gain [34]. Second, insufficient sleep has been associated with increased sympathetic activity, including increased levels of catecholamines and cortisol [35]. Third, sleep deprivation increases inflammation [36]. Inflammatory markers, including C-reactive protein, interleukin-6, and tumor necrosis factor, are positively related to obesity and the aggravation of insulin resistance [37]. Fourth, sleep deprivation promotes intestinal bacterial translocation across the intestinal epithelium [38], thus causing breakdown of the intestinal epithelial barrier. Increased catecholamine and cortisol levels due to sleep loss result in the breakdown of the intestinal epithelial cell tight junctions [39]. Consequently, weakened tight junctions may provide the gut microbiota and their metabolites an opportunity to cause systemic inflammation [39], affecting the host’s immune system. Furthermore, a compromised balance between intestinal microbes and the host’s immune system can lead to systemic inflammation and insulin resistance [40]. Finally, poor sleep may have common genetic pathways with metabolic dysfunction [41]. These findings imply that NRS mediates the activity of the sympathetic nervous system, immune system, and hypothalamic–pituitary–adrenal system, resulting in metabolic dysfunction.

### Strengths and limitations

The strengths of this study are the large sample size, the consideration of changes in NRS status during the survey period, the detailed evaluation of potential confounders, and a long follow-up period. However, there are certain limitations. First, the NRS question in this study lacked reliability and validity. Although there are several scales for NRS, there is no standardized assessment tool available in Japan [9]. NRS may overlap with (or include) SDB, short sleep duration, and insomnia. Consequently, our results may reflect an association between sleep problems and MetS. However, the NRS measure in this study included the main symptom of unrefreshing sleep. Therefore, these findings may provide the necessary evidence for the association between NRS and MetS. Future studies should adopt standardized, valid, and reliable measures for NRS. Second, we could not access other sleep information, including sleep duration, insomnia symptoms, use of sleep aids, and SDB. However, a previous study showed that NRS prevalence was inversely proportional to sleep duration and that NRS is recognized as a symptom of SDB [8]. Therefore, NRS is considered a suitable index for comprehensive judgment of sleep problems. Moreover, future studies should evaluate sleep status using objective tools, such as an accelerometer or actigraph. Third, although we adjusted for potential confounding variables, information on socioeconomic status, educational level, quality of life, and mental health status was lacking. Although numerous studies have found an association between the abovementioned variables and NRS scores, future studies should consider these variables. Finally, selection bias may have affected this study. We excluded 31,095 participants because of insufficient NRS information. Therefore, we should consider the validity of the current results. The prevalence of NRS in the current study was higher than that of previous Japanese studies using the same NRS question [8]. It is still possible that this study’s participants were more likely to emphasize their NRS symptoms. In addition, we could not collect information from younger individuals in this study. The prevalence of NRS in younger populations was equivalent to that in the age group of 50–59 years in Japan [42], and long-term NRS exposure may be a risk factor for premature MetS. Future prospective studies should investigate the NRS status of younger populations. In addition, unhealthy individuals may not have undergone annual medical checkups because they visited medical institutions directly. Consequently, our findings may not be generalizable to younger individuals, those who are less aware of their health status, and those on a leave of absence or without an occupation.

## Conclusions

The findings of this large-scale cohort study conducted among middle-aged Japanese adults suggest that NRS is positively associated with the incidence of MetS. Despite the recognized importance of adequate sleep for well-being, most Japanese are not satisfied with their sleep. Therefore, the current results may help design more effective prevention strategies for MetS to ensure adequate amount and quality of sleep. Furthermore, in clinical settings, physicians, in collaboration with sleep experts, should assess sleep status when treating MetS and its components. Then, treatment and/or assessment of NRS scores can improve MetS. Consequently, our findings provide strong evidence to guide and develop sleep health policies.

## Supplementary Information

**Additional file 1: Table S1.** Associations between NRS and development of MetS according to sex. **Table S2. **Associations between NRS and development of obesity, hypertension, diabetes, and dyslipidemia according to sex.

## Data Availability

The data included in this article will be shared by the corresponding author upon reasonable request.

## Abbreviations

CI: Confidence interval
CVD: Cardiovascular disease
HDL: High-density lipoprotein cholesterol
HR: Hazard ratio
MetS: Metabolic syndrome
NRS: Nonrestorative sleep
SDB: Sleep-disordered breathing
TG: Triglycerides
