# Supplementary material for: Nonrestorative sleep is a risk factor for metabolic syndrome in the general Japanese population
Source: Diabetol Metab Syndr. 2023 Feb 21;15:26. doi: 10.1186/s13098-023-00999-x (PMC9942313; doi:10.1186/s13098-023-00999-x)
Supplement: Supplementary file 1 — Additional file 1: Table S1. Associations between NRS and development of MetS according to sex. Table S2. Associations between NRS and development of obesity, hypertension, diabetes, and dyslipidemia according to sex. [file 13098_2023_999_MOESM1_ESM.docx]

Additional Material

Additional file: Table S1. Associations between NRS and development of MetS according to sex

|  | MetS | | | | | | | | | | | | |
| --- | --- | --- | --- | --- | --- | --- | --- | --- | --- | --- | --- | --- | --- |
|  | Men | | | | | |  | Women | | | | | |
|  | HR | 95% CI | | | p-value | z |  | HR | 95% CI | | | p-value | z |
| NRS | 1.11 | 1.07 | - | 1.15 | < 0.001 | 5.5 |  | 1.26 | 1.12 | - | 1.42 | < 0.001 | 3.8 |

Adjusted for age, sex, smoking, heavy alcohol consumption, skipping breakfast, and non-regular exercise.

HRs and P-values were calculated using the Cox proportional hazards model.

MetS: waist circumference of ≥85 cm in men and ≥90 cm in women and two or more of the following: 1) blood pressure of ≥130/85 mmHg or use of antihypertensive medication; 2) TG level of ≥150 mg/dL, HDL-C level of <40 mg/dL, or use of antilipidemic medication; and 3) glucose level of ≥110 mg/dL or use of hypoglycemic medication

Additional file: Table S2. Associations between NRS and development of obesity, hypertension, diabetes, and dyslipidemia according to sex

|  | Obesity | | | | | |  | Hypertension | | | | | |
| --- | --- | --- | --- | --- | --- | --- | --- | --- | --- | --- | --- | --- | --- |
| Men | HR *^1^ | 95% CI | | | p-value | z |  | HR *^2^ | 95% CI | | | p-value | z |
| NRS | 1.22 | 1.16 |  | 1.28 | <0.001 | 7.9 |  | 1.07 | 1.04 |  | 1.11 | <0.001 | 4.0 |
| Women | HR *^1^ | 95% CI | | | p-value | z |  | HR *^2^ | 95% CI | | | p-value | z |
| NRS | 1.33 | 1.03 |  | 1.72 | 0.033 | 2.4 |  | 1.04 | 0.97 |  | 1.12 | 0.278 | 1.1 |
|  | Diabetes | | | | |  |  | Dyslipidemia | | | | |  |
| Men | HR *^3^ | 95% CI | | | p-value | z |  | HR *^4^ | 95% CI | | | p-value | z |
| NRS | 1.02 | 0.97 |  | 1.08 | 0.440 | 0.8 |  | 1.00 | 0.97 |  | 1.03 | 0.904 | -0.1 |
| Women | HR *^3^ | 95% CI | | | p-value | z |  | HR *^4^ | 95% CI | | | p-value | z |
| NRS | 1.12 | 0.96 |  | 1.30 | 0.148 | 1.5 |  | 1.04 | 0.98 |  | 1.09 | 0.206 | 1.3 |

*1 Adjusted for age, sex, smoking, heavy alcohol consumption, skipping breakfast, non-regular exercise, diabetes, hypertension, and dyslipidemia

*2 Adjusted for age, sex, BMI, smoking, heavy alcohol consumption, skipping breakfast, non-regular exercise, diabetes, and dyslipidemia

*3 Adjusted for age, sex, BMI, smoking, heavy alcohol consumption, skipping breakfast, non-regular exercise, hypertension, and dyslipidemia

*4 Adjusted for age, sex, BMI, smoking, heavy alcohol consumption, skipping breakfast, non-regular exercise, diabetes, and hypertension

HRs and P-values were calculated using the Cox proportional hazards model.

Obesity: BMI of ≥25 kg/m^2^

Hypertension: ≥140/90 mmHg or use of antihypertensive medication

Diabetes: ≥126 mg/dL, HbA1c level of 6.5%, or use of hypoglycemic medication
